# Characterizing semen abnormality male infertility using non-targeted blood plasma metabolomics

**DOI:** 10.1371/journal.pone.0219179

**Published:** 2019-07-05

**Authors:** Pan Ma, Zhimin Zhang, Xinyi Zhou, Jiekun Luo, Hongmei Lu, Yang Wang

**Affiliations:** 1 College of Chemistry and Chemical Engineering, Central South University, Changsha, PR China; 2 Department of Integrated Traditional Chinese and Western Medicine, Xiangya Hospital, Central South University, Changsha, PR China; University of Porto, PORTUGAL

## Abstract

Semen abnormality (SA) male infertility has become a worldwide reproductive health problem. The invasive tests (e.g., testicular biopsy) and labor-intensive methods of semen collection severely inhibit diagnosis of male infertility. In addition, the pathogenesis and biological interpretation of male infertility are still obscure. In this report, a total of 84 semen abnormality (SA) patients, diagnosed as teratozoospermia (TE, n = 21), asthenozoospermia (AS, n = 23), oligozoospermia (OL, n = 20), azoospermia (AZ, n = 20), and age-matched healthy controls (HC, n = 29) were analyzed by GC-MS for discrimination analysis and discovery of potential biomarkers. Twenty-three biomarkers were obtained by multivariate statistical method (partial least squares-discriminant analysis, PLS-DA) and univariate statistical method (analysis of variance, ANOVA) with comparisons of TE versus HC, AS versus HC, OL versus HC and AZ versus HC. Based on those biomarkers, the most relevant pathways were mainly associated with the metabolism of carbohydrates, amino acids, and lipids. The principal metabolic alternations in SA male infertility included increased levels of energy-related metabolisms, such as tricarboxylic acid cycle, pyruvate metabolism, glyoxylate and dicarboxylate metabolism, glycine, serine, threonine metabolism and saturated fatty acid metabolism. Furthermore, increased levels of glutathione metabolism were related to oxidative stress. Finally, decreased levels of arginine and proline metabolism and inositol phosphate metabolism were observed. In conclusion, blood plasma metabolomics is powerful for characterizing metabolic disturbances in SA male infertility. From metabolic pathway analysis, energy production, oxidation stress and the released enzyme during spermatogenesis take the primary responsibilities for SA male infertility.

## 1. Introduction

Infertility is a worldwide reproductive health problem and affects approximately 15% of couples [1]. Male infertility accounts for 60% of infertility problems [2]. Normal sexual function with pregnancy failure (the so-called semen abnormality, SA) is a common cause of male infertility [3]. According to sperm concentration, motility and sperm morphology, SA male infertility can be divided into different subgroups: teratozoospermia (TE), asthenozoospermia (AS), oligozoospermia (OL) and azoospermia (AZ), etc. Nowadays the diagnosis of male infertility frequently depends on the results of semen quality or requires more invasive surgical intervention [2]. Moreover, molecular mechanisms and biological interpretation of SA male infertility are still obscure [4,5]. Therefore, it is urgent to develop a noninvasive way to diagnose and elucidate the metabolic pathways and etiological mechanisms of SA male infertility.

Metabolomics is considered as a powerful systems biology approach dedicated to analyzing the global and dynamic alterations in metabolism. Metabolic profiling can detect comprehensive endogenous metabolites, including organic acids, amino acids, fatty acids, sugar, cholesterol and other substances by the analyzing body fluids (e.g., plasma) and tissue (e.g., testicular tissue). Combined with advanced analytical techniques (e.g., GC-MS, LC-MS and NMR) and high-throughput bioinformatics tools, non-targeted metabolomic approaches are being widely used for the analysis of male infertility.

Though the researches on male infertility have made great progress in different research systems, semen analysis (sperm concentration, motility, and morphology) is the most widely used diagnostic method for male infertility [6,7]. Although andrology clinics go to great length to reassure men who are attending fertility assessment and the vast majority of men are able to produce a semen sample, the collection of semen samples has an embarrassing, difficult and stressful experience for patients. For azoospermic or cryptozoospermic diagnosis, or for therapeutic purposes, some invasive procedures such as testicular biopsy and microdissection are also applied [8–10]. These invasive procedures may cause inflammatory changes, hematoma, parenchymal fibrosis, or a permanent devascularization of the testes [11]. Instead, urine, serum and plasma may be better potential biologic matrices as they are easy to acquire. Some studies have successfully applied urine, serum and plasma for the diagnosis of male infertility. Zhang et al. reported a urinary metabolomics method to the diagnosis of oligozoospermic infertility [12]. A serum metabolomics study is employed to explore significant difference of metabolite profiles among three groups of different sperm concentration (low, intermediate, high) [13]. Recently, our group comprehensively proposed plasma metabolomics method to differentiate fertile individuals from patients with erectile dysfunction and SA [3]. These studies have demonstrated that urine, serum and plasma are suitable for the analysis of male infertility.

The molecular mechanism of male infertility plays an important role in the cause and treatment of male infertility. Some studies have provided some explanation of the cause of male infertility. Varicocele and oxidative stress (OS) were found to be associated with male infertility. Studies have shown that the presence of varicocele appeared to negatively affect sperm density, motility, and morphology by semen analysis [14–16]. Studies on the metabolic profiling of OS in seminal plasma indicate that OS has a great influence on male infertility [10,17]. Further studies showed OS can result in sperm DNA damage, which is related to male infertility [18,19]. However, molecular mechanism of varicocele and OS is unclear. Recently, a blood plasma metabolomics analysis of erectile dysfunction and semen abnormality was reported and some biomarkers were identified [3]. However, no further metabolic pathway analysis (MPA) was employed for the identified biomarkers. Additionally, systematic metabolic pathway analysis was applied to normozoospermic infertile men in a urine metabolomics [20]. By the MPA, the pathways of energy production, antioxidation, and hormone regulation in spermatogenesis were explained for oligozoospermic infertility. From this study, metabolic pathway analysis can provide more explanation of molecular mechanism to male infertility. Thus far, no comprehensive metabolomics study has reported a complete MPA for TE, AS, OL and AZ, simultaneously.

In this study, a blood plasma metabolomics method based on GC-MS, PLS-DA and MPA, was proposed to characterize the metabolic features of SA male infertility. The objective of this study was to investigate whether the blood plasma metabolomics can be used to differentiate healthy controls (HC) from TE, AS, OL, AZ. Then, we further investigated the potential biomarkers and metabolic pathways for deciphering the pathogenesis of SA male infertility.

## 2. Materials and methods

### 2.1 Chemicals

High-performance liquid chromatography (HPLC) grade methanol was purchased from the Tedia Company (Inc., Fairfield, USA). BSTFA+1% TMCS (N, O-bis (trimethylsilyl) trifluoroacetamide with 1% trimethylchlorosilane for GC) (> 99.0% purity), methoxyamine hydrochloride (> 98.0% purity) and pyridine (> 99.8% purity), internal standard 2-isopropylmalic acid (> 98.0% purity) and the other 25 chemical standards of metabolites (S1 Table) were commercially obtained from Sigma-Aldrich (St. Louis, MO, USA).

### 2.2 Sample collection

All subjects were volunteers sequentially recruited from Xiangya Hospital of Central South University from June 2015 to December 2015. Written informed consents were obtained from all participants and every participant was also informed about the purpose of this research. This study was approved by the Medical Ethics Committee of Xiangya Hospital. The clinical study was conducted in accordance with the principles of the Declaration of Helsinki. The infertile men were collected from the men who attended the Xiangya Hospital because of conception failure for at least 12 months. A questionnaire was employed to collect information including age, smoking and drinking history, metabolic disease, genetic disease, medical history and sexual and reproduction status. To exclude the influence of confounding factor contributing to male infertility, the subject was excluded from the study if any of the following conditions were detected during routine work-up for male infertility: 1) current history of abuse of drugs or alcohol; 2) metabolic diseases (diabetes, coronary heart disease, hyperlipidemia) and urogenital diseases; 3) hormonal treatments and medical history of infertility risk factors (varicocele, vasectomy, and orchidopexy); 4) other known causes (genetic disease, infection, occupational exposure to the agents) related to male infertility. The fertile men whose partner had a baby or pregnant within the previous year were recruited and sampled as health control during the same period.

Referring to the fifth edition World Health Organization (WHO) Laboratory Manual for the Examination and Processing of Human Semen [21], TE was defined as sperm morphology (normal form) < 4%; AS was defined as progressive motility < 32%; OL was defined as total sperm number < 39 million per ejaculate; AZ was defined as total sperm number = 0. Ultimately, 113 individuals were divided into five groups. Four SA subgroups include TE (n = 21, age: 28.98±4.80), AS (n = 23, age: 28.85±5.38), OL (n = 20, age: 28.78±5.62) and AZ (n = 20, age: 28.60±4.93). Twenty-nine fertile men (age: 28.07±4.51) were defined as HC. Statistical analysis of age, smoking, and drinking among SA subgroups and HC are presented in S2 Table. No significant differences are observed for age, smoking, and drinking. Blood sample of all participants was collected after overnight fasting. Each individual had a venous blood sample drawn. The blood was collected into heparinized tubes and centrifuged at 4000 × g and 4°C for 20 min. The blood plasma was separated and kept frozen at -80°C until analysis.

### 2.3 Sample preparation

Each 100 μL blood plasma sample was mixed with 300 μL methanol to precipitate the protein. Then, 30 μL of internal standard (2-isopropylmalic acid/methanol, 1 mg/mL) was added and mixed. Next, the mixture was vortex-mixed for 15 s and centrifuged for 10 min (16,000 rpm, 4°C). The supernatant (330 μL) was transferred into a 5 mL glass centrifugation tube and evaporated to dryness by N_2_ gas. Next, 50 μL methoxyamine/pyridine (20 mg/mL) was added to the dry tube, and the resultant mixture was mixed on a vortex for 30 s and incubated for 1 h at 70°C with a glass plug. Finally, 100 μL of BSTFA derivatization agent was added to the residue, vortex-mixed for 30 s, and heated in a water bath at 70°C for 1 h with a glass plug. The final solution was taken for GC-MS analysis. Importantly, after being processed, all samples were analyzed with GC-MS in a random order in order to avoid the run order effect. Quality control (QC) samples were prepared by pooling 35 μL aliquots from each blood plasma sample and vortex mixing (5 min). Sample preparation for QC samples was performed in the same way as the sample preparation described above.

### 2.4 Metabolic profiling analysis

The plasma metabolic profiling analysis was conducted on a Shimadzu GC 2010 gas chromatography instrument coupled to a Shimadzu QP2010 mass spectrometer (Shimadzu, Japan), and equipped with an autosampler GL 221–34618. The column used for all analyses was an Agilent DB-5MS with a deactivated fused silica column (30 m × 0.25 mm × 0.25 μm). The column temperature procedure was designed as follow: initially maintained at 70°C for 4 min, programmed to 300°C at a rate of 8°C/min, and then held at 300°C for 3 min. Helium was used as a carrier gas with a flow rate of 1.0 mL/min. The septum purge was turned on with a flow rate of 3 mL/min the entire time. The injector temperature, the interface temperature and the ion source temperature were set at 280°C, 250°C and 200°C, respectively. The mass spectrometer was operated under electron impact (EI) in full scan mode over a range from m/z 35 to 800 with a 0.2 s scan velocity, and the detector voltage was 0.96 kV. Ionization was achieved by a 70 eV electron beam.

To ensure data quality of metabolic profiling, 11 QC samples were run before analyzing the sample sequence. In each batch, the injection sequence was 1 QC—6 subject samples—1 blank—1 QC—6 subject samples—1 blank—1 QC. Therefore, each batch was composed of 12 subject samples, 3 QC samples and 2 blank samples. The QC samples were used to calculate technical precision and for signal correction.

### 2.5 Data processing and analysis

The raw data files (.D format) were converted to the NetCDF format, which can be imported into the MS-assisted resolution of signal (MARS) software package [22]. The qualitative and quantitative table was obtained using the MARS software package based on multivariate resolution methods. All of the detected peak features were identified by standards and the NIST Mass Spectral Search Program (Version 2.0).

**Fig 1:**
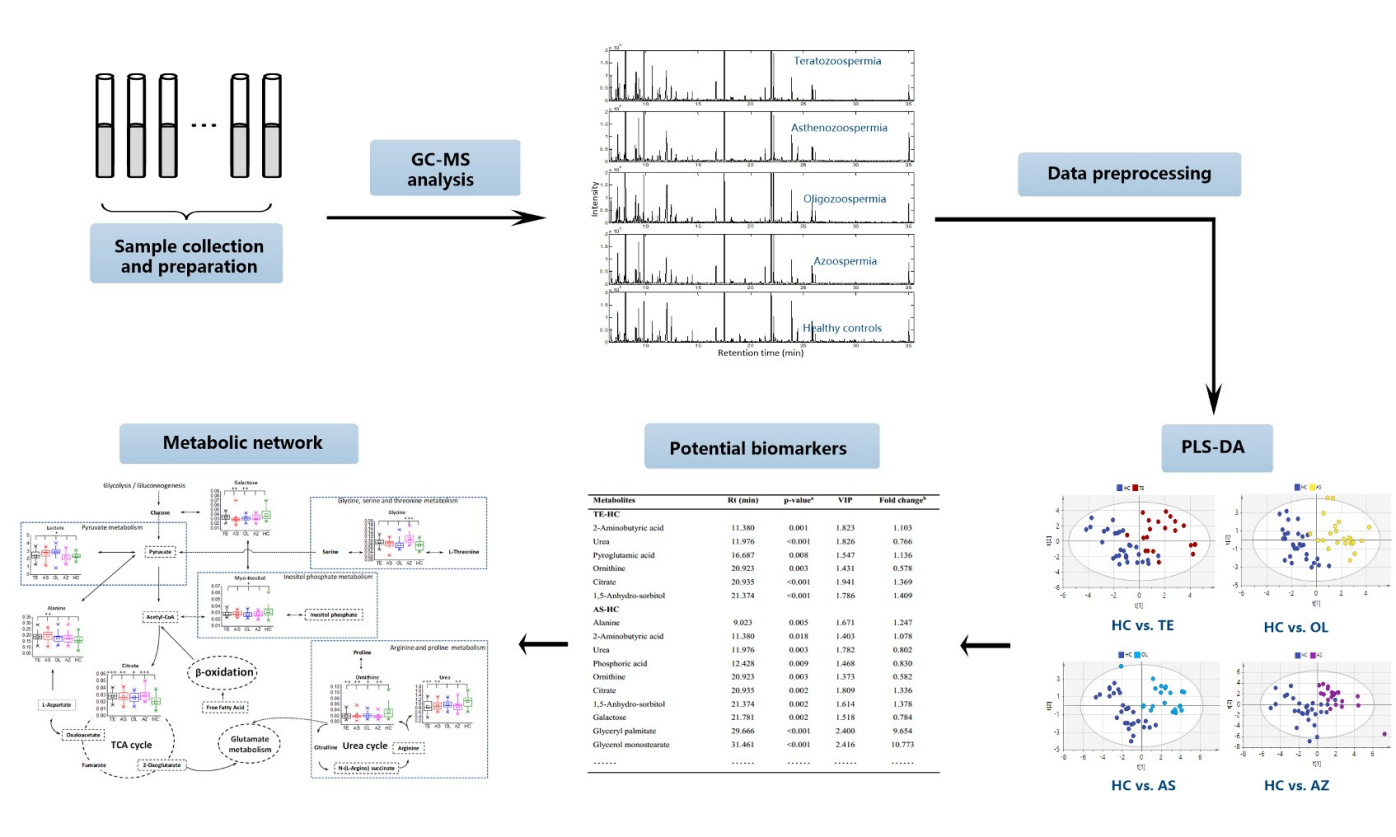
The workflow of metabolic analysis of SA male infertility.

Statistical significance was calculated using analysis of variance (ANOVA) with Dunnett post-hoc analysis (p-value < 0.05) as implemented in the SPSS version 24 for windows software package. The peak area table for all metabolites was imported into the SIMCA-P program (version 14.1, Umetrics) for multivariate analysis. Principal component analysis (PCA) and partial least squares discriminate analysis (PLS-DA) were applied with unit variance (UV) scaling. The parameters of the models, such as the R^2^X, R^2^Y, Q^2^Y, area under the receiver operating characteristic curve (AUC) and permutation test were calculated to evaluate the quality of the multivariate models and avoid the risk of overfitting. Hierarchical cluster analysis (HCA) was conducted using the MeV software package (version 4.9). Moreover, the metabolic pathway analysis web tool, including MetPA (http://metpa.metabolomics.ca) [23] and the Kyoto Encyclopedia of Genes and Genomes (KEGG, http://www.genome.jp/kegg/) [24], were employed to identify the potentially disordered metabolic pathways. The workflow of metabolic analysis of SA male infertility is shown in Fig 1.

## 3 Results

### 3.1 Metabolic profiling of blood plasma samples

In this study, all blood plasma samples were profiled by GC-MS. The typical ion chromatograms (TIC) of blood plasma metabolic profiling for TE, AS, OL, AZ and HC are shown in the supplementary materials (S1 Fig). As shown in S1 Fig, the species of metabolites are similar among 5 groups, but the contents of metabolites are different. The features of retention time, pure chromatographic profiles, mass spectra, and peak areas were obtained from MARS software package. As shown in S1 Table, 53 metabolites, including amino acids, carbohydrates, lipids, organic acids and urea, were identified by a NIST search. The R-match was used to express the similarity between the extracted mass spectrum and the spectrum in the NIST library. Moreover, 25 metabolites were unambiguously identified and validated by the authorized chemical standards. Before statistical analysis, all peak areas of corresponding metabolites in each sample were normalized to peak area of the internal standard. A QC strategy was always applied to assure the data reliability. According to the US Food and Drug Administration, the RSD (relative standard deviation) value allows up to 30% for biomarkers discovery [25]. The RSD of the QC samples was calculated. From S1 Table, only three of them are over 30%. After removing these 3 metabolites with low reproducibility, a 2D matrix (113 samples × 50 variables) was generated and exported to conduct the statistical analysis.

### 3.2 Statistics analysis

**Fig 2:**
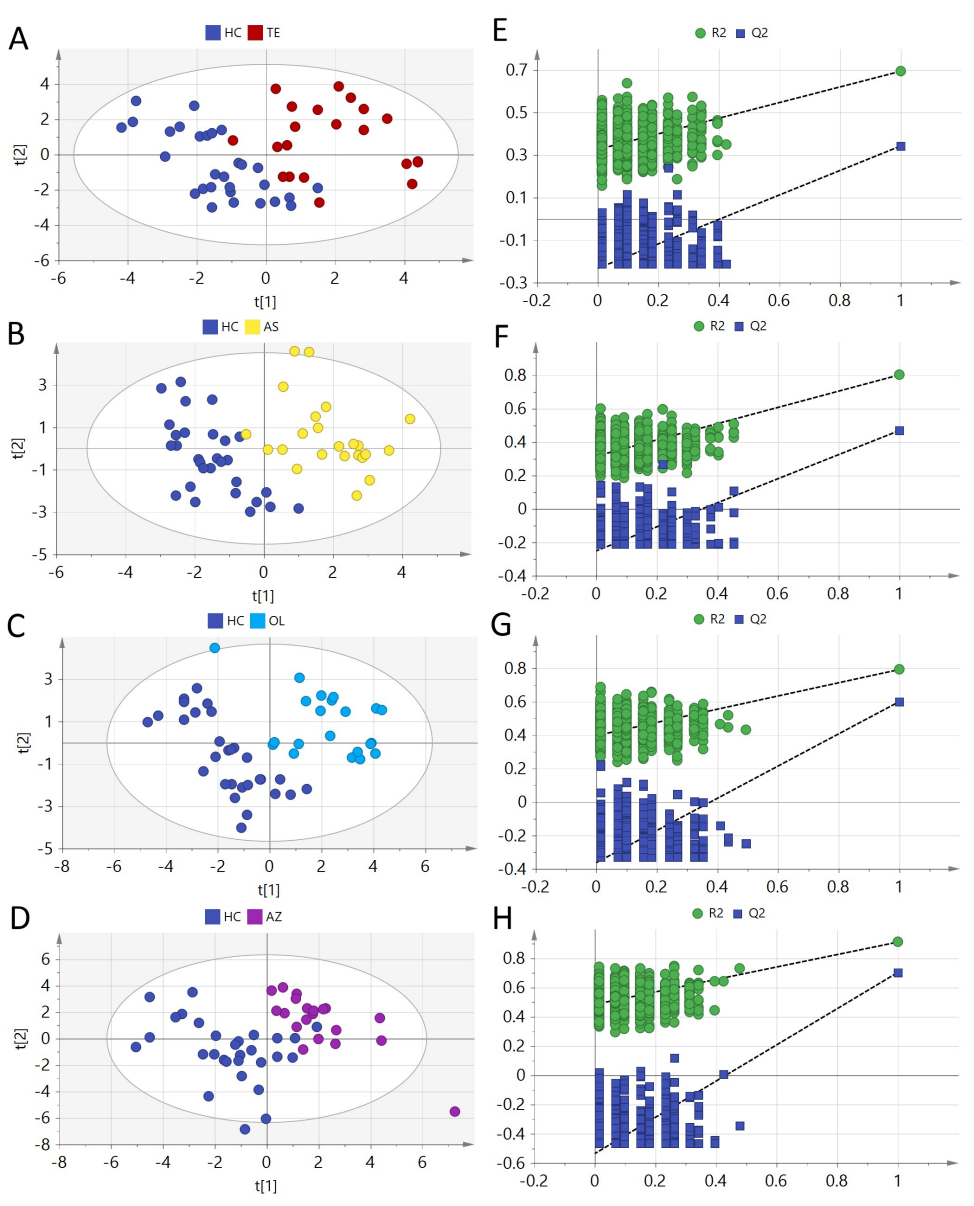
PLS-DA score plots of (A) TE vs. HC, (B) AS vs. HC, (C) OL vs. HC and (D) AZ vs. HC; the corresponding random permutation test results (n = 900) of the PLS-DA models (E, F, G and H).

PCA is a typical unsupervised pattern recognition technique and can project the matrix data (113 samples × 50 variables) into a lower dimensional space. From the score plot (S2 Fig) of PCA model (R^2^X = 0.879), 4 SA subgroups and HC are totally overlapped. However, a clear cluster of the pooled QC samples indicated that the sample analysis sequence had satisfactory stability and repeatability. For each metabolite, the p-value was calculated using ANOVA with Dunnett post-hoc analysis between 4 SA subgroups and HC (S3 Table). A p-value less than 0.05 indicates a significant difference. PLS-DA was applied to the supervised classification of the TE, AS, OL, AZ and HC. Parameters of the PLS-DA model are as follows: TE-HC (R^2^X = 0.407, R^2^Y = 0.764, Q^2^ = 0.361, AUC = 0.995), AS-HC (R^2^X = 0.418, R^2^Y = 0.901, Q^2^ = 0.437, AUC = 0.998), OL-HC (R^2^X = 0.518, R^2^Y = 0.911, Q^2^ = 0.704, AUC = 0.999), and AZ-HC (R^2^X = 0.487, R^2^Y = 0.792, Q^2^ = 0.6, AUC = 0.998). From those parameters, 4 PLS-DA models show good ability to explain the raw matrix. Four SA subgroups show a clear separation from HC in the score plot (Fig 2A, 2B, 2C and 2D). From Fig 2E, 2F, 2G and 2H, the calculated R^2^ and Q^2^ values in permutation tests are lower than the original ones and the Q^2^ intercept on the vertical axis was less than zero. Therefore, the model is considered valid. The high AUC value (> 0.99) showed good model predictive performance. The variable importance in the projection (VIP) value was used to evaluate the importance of metabolites in PLS-DA model. The results are shown in S4 Table.

### 3.3 Discovery of candidate biomarkers

**Fig 3:**
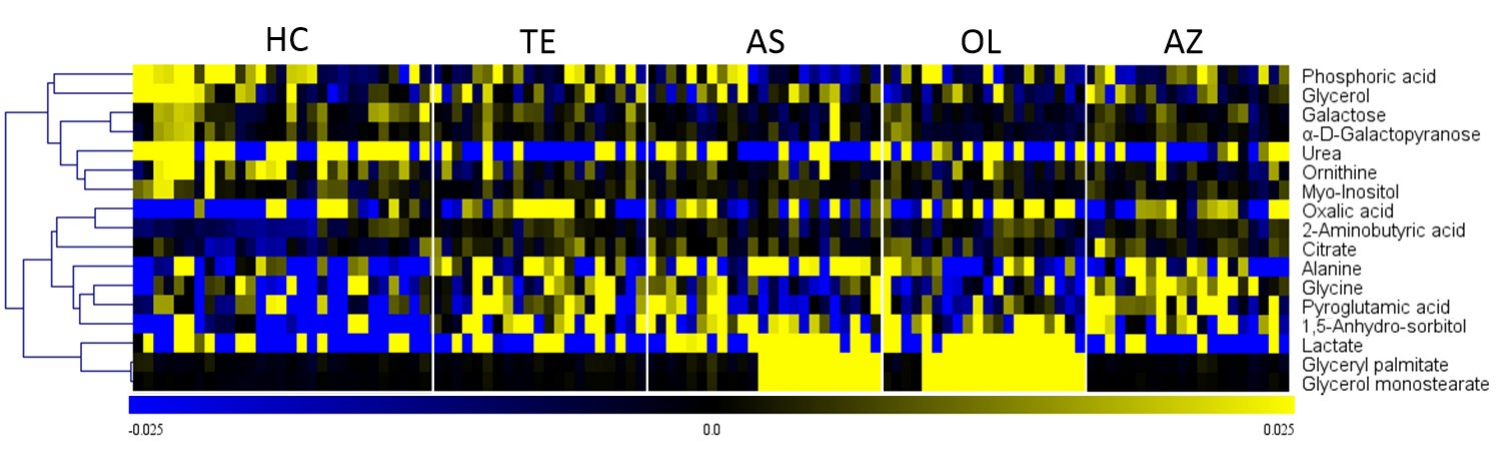
Heat map of the 17 biomarkers between TE, AS, OL, AZ and HC. The colors from blue to yellow indicate the relative contents of metabolites from low to high.

Biomarker discovery is the critical step for metabolomics studies. Selection of the informative metabolites is of great importance for metabolic pathways analysis and biological interpretation. The VIP value from PLS-DA model indirectly reflects the correlation of metabolites with disease [26]. Four biomarker panels with VIP more than 1.00 and p-value of ANOVA less than 0.05 are shown in Table 1. Four biomarker panels include a total of 34 metabolites containing 17 different metabolites. Those 17 biomarkers can well differentiate 4 SA subgroups (TE, AS, OL and AZ) from HC. HCA was performed according to the Pearson correlation coefficients of metabolic levels of 17 biomarkers. As shown in Fig 3, it is apparent that the metabolic levels of biomarkers in HC are quite different from those in 4 SA subgroups.

**Table 1:** Statistical analysis of biomarkers based on PLS-DA and ANOVA between 4 SA subgroups and HC respectively.

**Metabolites**	**Rt (min)**	**p-value**^a^	**VIP**	**Fold change**^b^
**TE-HC**				
2-Aminobutyric acid	11.380	0.001	1.823	1.103
Urea	11.976	<0.001	1.826	0.766
Pyroglutamic acid	16.687	0.008	1.547	1.136
Ornithine	20.923	0.003	1.431	0.578
Citrate	20.935	<0.001	1.941	1.369
1,5-Anhydro-sorbitol	21.374	<0.001	1.786	1.409
**AS-HC**				
Alanine	9.023	0.005	1.671	1.247
2-Aminobutyric acid	11.380	0.018	1.403	1.078
Urea	11.976	0.003	1.782	0.802
Phosphoric acid	12.428	0.009	1.468	0.830
Ornithine	20.923	0.003	1.373	0.582
Citrate	20.935	0.002	1.809	1.336
1,5-Anhydro-sorbitol	21.374	0.002	1.614	1.378
Galactose	21.781	0.002	1.518	0.784
Glyceryl palmitate	29.666	<0.001	2.400	9.654
Glycerol monostearate	31.461	<0.001	2.416	10.773
**OL-HC**				
Oxalic acid	7.359	0.008	1.225	1.136
Lactate	8.090	0.010	1.395	1.218
2-Aminobutyric acid	11.380	0.015	1.186	1.083
Glycerol	12.454	0.037	1.064	0.745
Citrate	20.935	0.027	1.391	1.270
1,5-Anhydro-sorbitol	21.374	<0.001	1.538	1.430
Galactose	21.781	0.005	1.253	0.794
α-D-Galactopyranose	23.551	0.018	1.146	0.817
Myo-Inositol	24.296	0.023	1.003	0.849
Glyceryl palmitate	29.666	<0.001	2.514	13.975
Glycerol monostearate	31.461	<0.001	2.530	15.987
**AZ-HC**				
Glycine	9.461	<0.001	1.819	1.425
2-Aminobutyric acid	11.380	0.009	1.269	1.087
Urea	11.976	0.004	1.472	0.797
Pyroglutamic acid	16.687	<0.001	1.656	1.186
Ornithine	20.923	0.002	1.471	0.541
Citrate	20.935	<0.001	1.795	1.409
1,5-Anhydro-sorbitol	21.374	0.002	1.532	1.403

a: p-value of ANOVA with Dunnett post-hoc test.

b: relative metabolite content in SA subgroups compared with HC.

Four biomarker panels were different with each other. It revealed that there may be difference among 4 subgroups. Six cross-comparisons among TE, AS, OL and AZ were employed to determine if there is a difference. As shown in the S3 Fig, the poor separation and small value of the model parameters (R^2^X, R^2^Y, Q^2^) demonstrated that the identified metabolites could not discriminate TE, AS, OL and AZ. This suggests that one analytical technique (GC-MS) is not enough to characterize the small metabolic difference among those subgroups. Other complementary analytical techniques, such as LC-MS, NMR and CE-MS, are needed to find and identify some additional biomarkers for discriminating the SA subgroups. In addition, the small number of samples in the current study may cause the metabolic difference to be covered by individual differences. Based on those reasons, four biomarker panels would be used for MPA and 4 subgroups were treated as one SA group for the discussion section.

### 3.4 Metabolic pathway analysis

**Fig 4:**
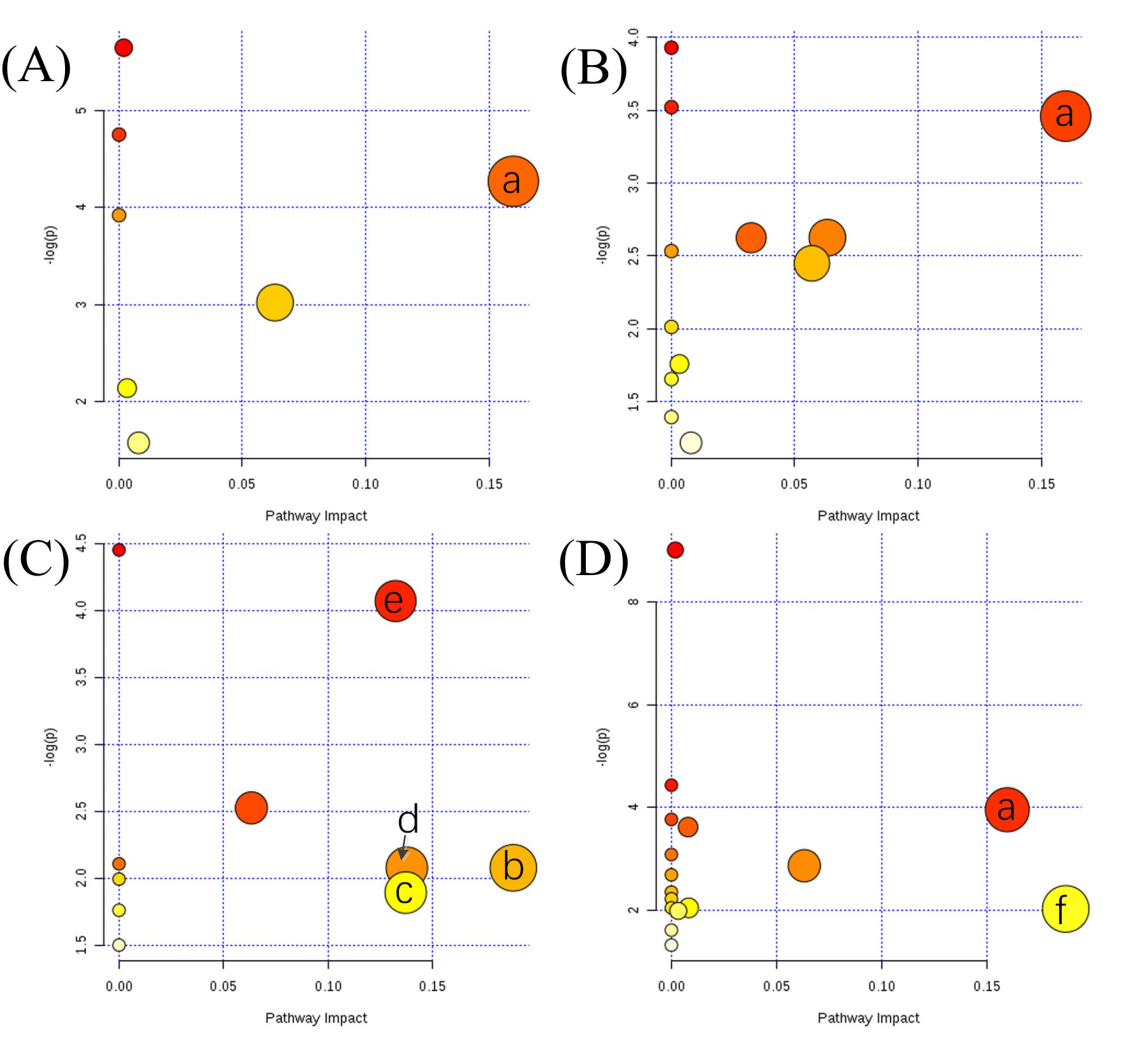
Summary of altered metabolic pathways analysis with MetPA when comparing (A) TE to HC, (B) AS to HC, (C) OL to HC, and (D) AZ to HC. a: arginine and proline metabolism; b: glycerolipid metabolism; c: inositol phosphate metabolism; d: pyruvate metabolism; e: glyoxylate and dicarboxylate metabolism; f: glycine, serine and threonine metabolism.

**Fig 5:**
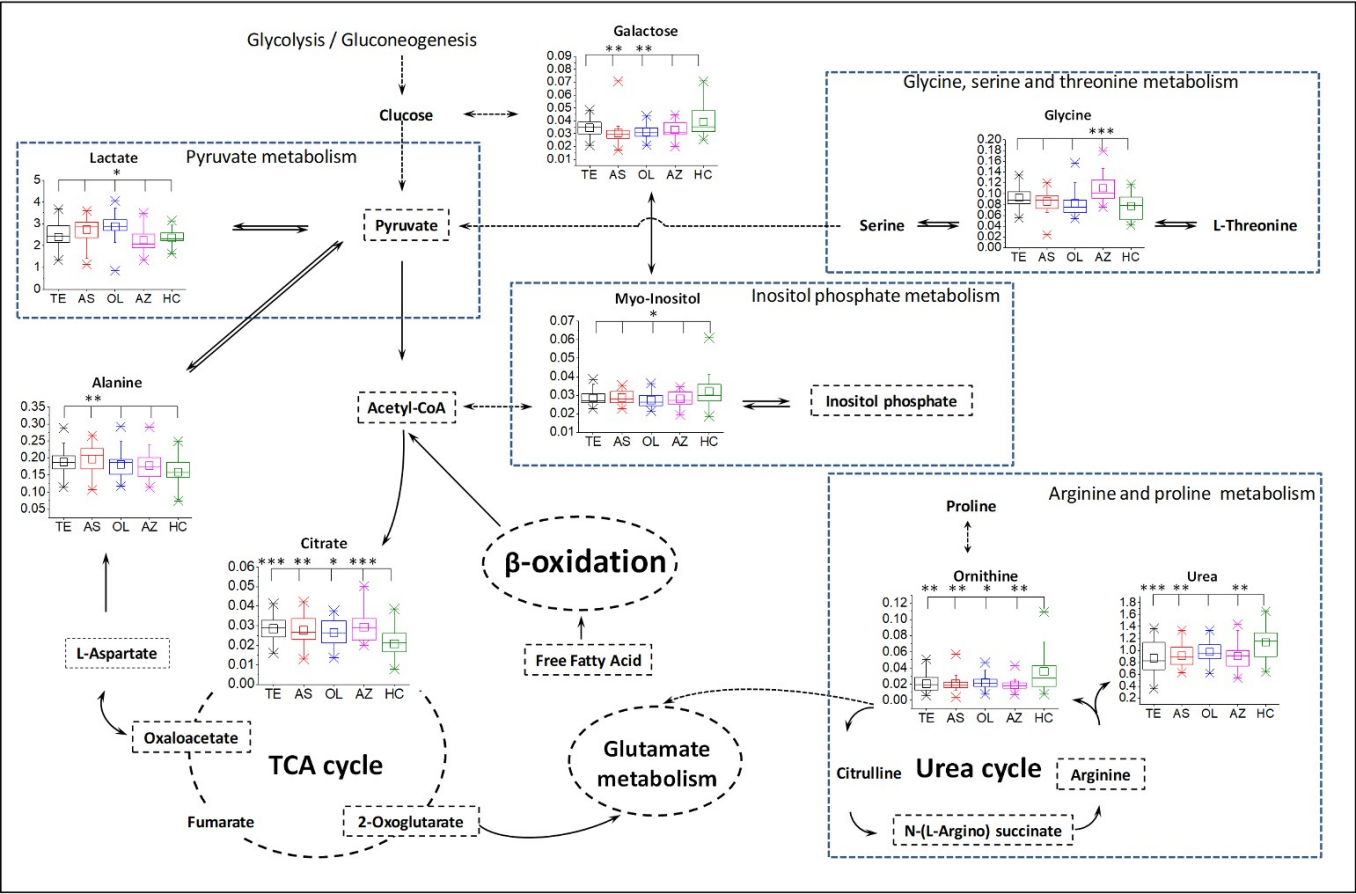
Metabolic network of the significantly changed metabolites. All the p-values between SA subgroups and HC were calculated using ANOVA with Dunnett post hoc. *, p-value<0.05; **, p-value<0.01; ***, p-value<0.001. Nonsignificantly changed metabolites are shown only by name, and the names with a dashed rectangle represent the undetected metabolites or metabolisms. Dashed arrows indicated multistep reactions. Single and double bidirectional arrows indicate reactions with the same conditions and different conditions, respectively. The dashed circle denoted the cycle of reaction.

**Fig 6:**
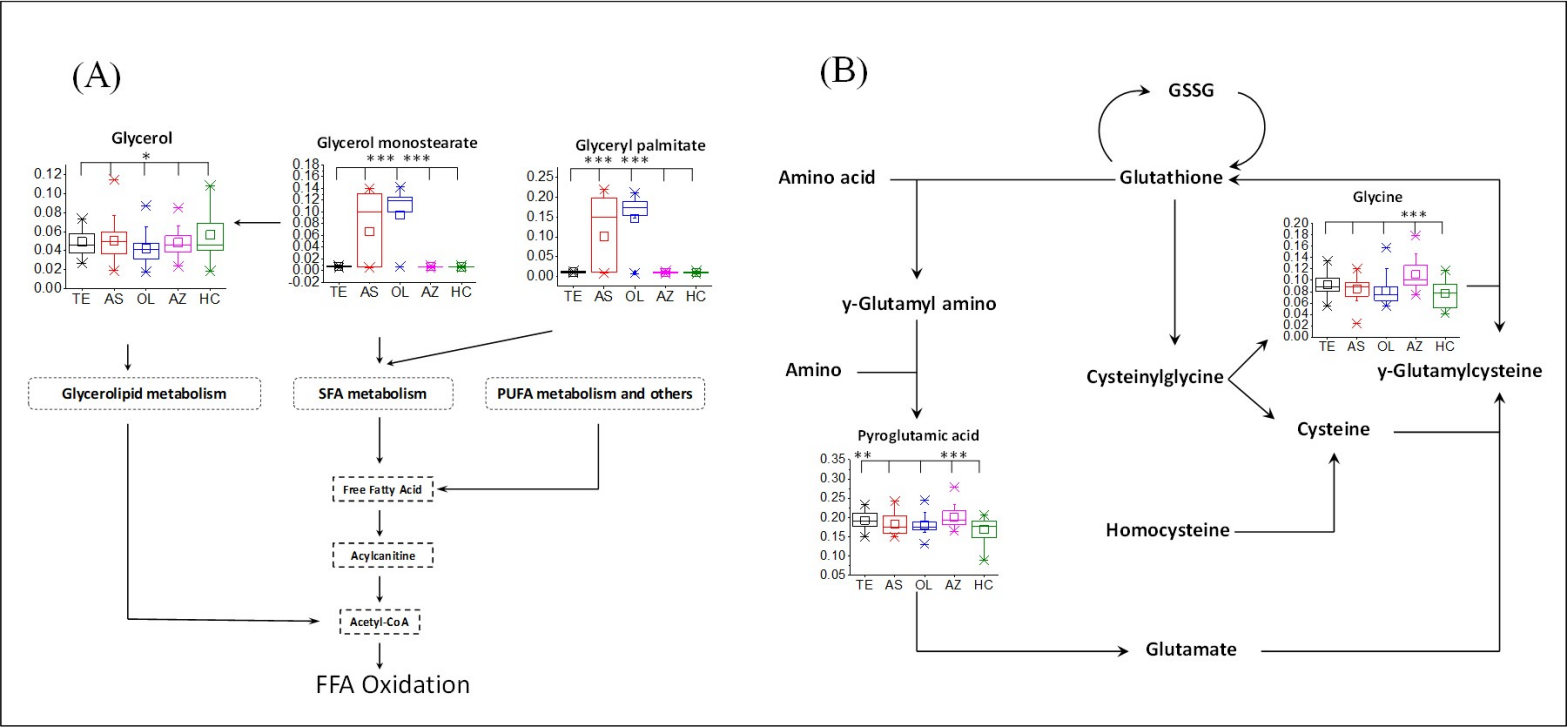
The overview of lipid metabolism and glutathione metabolism. (A) lipid metabolisms; (B) glutathione metabolism. SFA: saturated fatty acid; PUFA: polyunsaturated fatty acid.

In order to fully explore the metabolic disorder between SA subgroups and HC, two MPA methods were used. Four biomarker panels (Table 1) were imported into MetPA for MPA, respectively. As shown in Figs 4 and 6 metabolic pathways with pathway impact higher than 0.1 are screened out, including arginine and proline metabolism (APM), glycerolipid metabolism (GLM), inositol phosphate metabolism (IPM), pyruvate metabolism (PM), glyoxylate and dicarboxylate metabolism (GDM), and glycine, serine and threonine metabolism (GSTM). As a supplement, 17 biomarkers (shown in Fig 3) were analyzed by KEGG and 2 metabolic pathways were identified, including glutathione metabolism (GM), and tricarboxylic acid cycle (TCA). Based on knowledge of the selected biomarkers and disordered metabolisms, a map of the SA male infertility-related metabolic pathways was constructed (Fig 5).

Metabolic characteristics associated with SA male infertility are revealed in Fig 5. The most relevant pathways are mainly associated with the metabolism of amino acids, lipids and carbohydrates. Several carbohydrate metabolic pathways were modified in SA male infertility, such as an increase in PM (including lactate), GDM (including oxalic acid, citrate and glycine), TCA (including citrate) and a decrease in IPM (including myo-inositol). In terms of amino acid metabolism, GM (including pyroglutamic acid and glycine) and GSTM (including glycine) increased in SA groups. Those disrupted amino acid metabolic pathways implied a wide range of abnormal functions within the body. The change in lipid metabolism was not the same as that of amino acids. The levels of two saturated fatty acid (SFA) ester, including glyceryl palmitate and glycerol monostearate, increased in SA male infertility. Besides, the level of glycerol in GLM decreased in SA male infertility. It can be inferred that the SA male infertility was also affected by lipid homeostasis.

## 4. Discussion

To our knowledge, there is a lack of published literature regarding the application of blood plasma metabolomics in characterizing male infertility with the teratozoospermia, asthenozoospermia, oligozoospermia and azoospermia. This is the first systematic study of the metabolism of 4 SA male infertility. Based on knowledge of the selected biomarkers and disordered metabolic pathways, some important metabolic signatures are shown in Figs 5 and 6. The latent relationships between disturbed metabolic pathways and SA male infertility will be discussed for the purpose of investigating the pathogenesis.

Spermatogenesis is highly energy-dependent, but the energy requirements to sustain normal sperm production are not reached in male infertility [12,27]. From MPA, most disordered pathways in this study are related to energy supply, such as glycolysis-related metabolisms, amino acid metabolisms and lipid metabolisms. Those metabolisms are upregulated for more energy during spermatogenesis. PM is a step of glycolysis and can convert pyruvate into acetyl-CoA for TCA. Lactate was identified as the biomarker for AS and OL in PM. Previous research showed that pyruvate and lactate are metabolized to produce ATP and are good energy substrates for sperm survival and movement [27,28]. Elevated level of citrate is tightly associated with energy production via the TCA by fatty acid β-oxidation in the mitochondria. Because of this increased catabolism, the level of amino acids and lipids consequently increase to satisfy the large energy demand during spermatogenesis. Glycine, a glucogenic amino acid, plays an important role in GDM and SGTM. It can be converted to glucose for more energy. The upregulation of fatty acid β-oxidation, including saturated fatty acid (SFA) and polyunsaturated fatty acid (PUFA), leads to excessive consumption of glycerol and free fatty acids (FFA). This leads to the decreased level of glycerol in GLM. Lipid catabolism always produces glycerol and free fatty acids. Therefore, lipids are needed to be upregulated for lipid catabolism. The increased levels of lipids (glycerol monostearate and glyceryl palmitate) shown in Fig 6A confirmed this. From those results, we can see that the revealed high energy requirements in SA male infertility are needed for spermatogenesis.

In addition to energy metabolism, oxidative stress (OS) is an important cause of male infertility [18,19]. Free radicals and reactive oxygen species (ROS) are detrimental to spermatozoa and lead to damage of the DNA [29,30]. Our observations indicated that SA male infertility is associated with aberrations in amino acid metabolism and lipid metabolism. Because oxidative damage is the primary driver for spermatozoa apoptosis, the level of antioxidative metabolites would be accordingly elevated. Glycine is a precursor substance of glutathione in GM. Glutathione plays important roles in antioxidant defense [31]. As shown in the GM cycle (Fig 6B), the striking elevation of glycine and pyroglutamic acid suggested an increased level of glutathione. Several studies have been shown that glutathione helps to protect sperm through reducing oxidative stress [19] and glutathione supplementation has a very positive effect on sperm morphology and motility [32,33]. In terms of lipid metabolisms, SFA and PUFA are always obtained from increased lipid catabolism. PUFA may play a key role in linking oxidative stress because the PUFA in the sperm plasma membrane is sensitive to lipid peroxidation [18,34,35]. The direct interaction of SFAs on SA male infertility is currently unclear. However, research studies have shown that the high intake of SFAs is positively linked to asthenozoospermia [36] and the high level of SFAs from dairy food intake has a detrimental effect on semen quality [37].

Myo-inositol, alanine, lactate, ornithine and urea were also identified as biomarkers. Those metabolites are related to infertility by other special functions. IMP plays a very important role in metabolic regulation, message transduction and various physiological functions of cells. A study has shown that myo-inositol could increase sperm motility and the number of spermatozoa retrieved after swim-up [38]. Moreover, that improvement was considered to associate with improved sperm mitochondrial function. Some enzymes were released during spermatogenesis, including alanine aminotransferase (ALT) and lactate dehydrogenase (LDH). Studies indicate that LDH can cause damages in sperm, which lead to male infertility [39]. Furthermore, the activity of ALT and LDH have an important effect on the quality of the sperm. The higher the quality of sperm, the lower the activity of ALT and LDH [40,41]. Therefore, the disorder of ALT and LDH may be the reason for the disorder of alanine and lactate. The urea cycle (UC) is a part of APM. Decreased levels of ornithine and urea in the UC cause a low level of arginine. Research has shown that arginine was required for sperm production, and arginine supplementation can help to boost healthy sperm count and improve fertility [42].

From those discussions, male infertility has a great metabolism change. Energy production, oxidative stress and a variety of special biological functions are identified to be related to male infertility. From metabolic disorder, it was suggested that dieting may help to the treatment of male infertility by elevating levels of important amino acids and nutrients associated with fertility [43].

## 5. Conclusions

In summary, the metabolic profiling analysis of blood plasma samples provided a holistic view of the metabolic features of SA male infertility. It confirmed that blood plasma metabolomics can be used to differentiate TE, AS, OL, AZ from HC. The biomarkers were filtered and identified between SA subgroups and HC. SA male infertility is mainly associated with the metabolism of carbohydrates, amino acids and lipids. Energy-related metabolisms, such as pyruvate metabolism, TCA and fatty acid β-oxidation, are also upregulated for energy supply to satisfy high energy demands during spermatogenesis. Moreover, oxidative stress is an important cause of SA male infertility. The antioxidative metabolites would be elevated to protect sperm from oxidative damage. Finally, the enzymes released during spermatogenesis such as alanine aminotransferase and lactate dehydrogenase may lead to metabolic disorders. Those metabolic features may be important for elucidating the etiological mechanism and therapy of SA male infertility.

## Supporting information

S1 Table Metabolite identification results of SA subgroups and HC. (DOCX)

S2 Table Statistical analysis of age, smoking and drinking among SA subgroups and HC. (DOCX)

S3 Table The p-value of ANOVA with Dunnett post-hoc test. (DOCX)

S4 Table The VIP value of PLS-DA models. (DOCX)

S1 Fig The typical total ion chromatograms of HC, TE, AS, OL and AZ blood plasma samples. (TIF)

S2 Fig Score plot of principal component analysis among TE, AS, OL, AZ and HC. (TIF)

S3 Fig Six cross-comparisons of PLS-DA among TE, AS, OL and AZ. The R^2^X, R^2^Y and Q^2^ of six models: a, 0.197, 0.513, -0.167; b, 0.253, 0.722, 0.234; c, 0.258, 0.399, -0.21; d, 0.241, 0.398, 0.047; e, 0.284, 0.492, 0.071; f, 0.323, 0.334, 0.131.
(TIF)

S1 File Qualitative and quantitative table used for the PLS-DA model (after normalization). (XLSX)
