# Supplementary material for: Characterizing semen abnormality male infertility using non-targeted blood plasma metabolomics
Source: PLoS One. 2019 Jul 5;14(7):e0219179. doi: 10.1371/journal.pone.0219179 (PMC6611580; doi:10.1371/journal.pone.0219179)
Supplement: S1 Table — (DOCX) [file pone.0219179.s001.docx]

S1 Table. Metabolite identification results of SA subgroups and HC.

| **Metabolites** | **t_R_(min)** | **R-match** | **RSD (%)** | **KEGG** | **HMDB** | **Pathway** |
| --- | --- | --- | --- | --- | --- | --- |
| Oxalic acid | 7.359 | 860 | 21.23 | C00209 | HMDB02329 | Glyoxylate and dicarboxylate metabolism |
| Lactate^a^ | 8.090 | 964 | 12.79 | C00186 | HMDB00190 | Pyruvate metabolism |
| Alanine^a^ | 9.023 | 927 | 18.34 | C00041 | HMDB00161 | Alanine, aspartate and glutamate metabolism |
| Glycine^a^ | 9.461 | 922 | 16.91 | C00037 | HMDB00123 | Glycine, serine and threonine metabolism |
| α-hydroxyisobutyric acid | 9.519 | 845 | 16.35 | C05984 | HMDB33721 | Propanoate metabolism |
| N-acetylglycine | 9.659 | 729 | 11.27 | ­- | HMDB00532 | ­- |
| Acetic acid | 9.972 | 767 | 32.74^b^ | C00033 | HMDB00042 | Pyruvate metabolism |
| β-hydroxyisobutyric acid^a^ | 10.244 | 927 | 11.56 | C01089 | HMDB00357 | Butanoate metabolism |
| Valine^a^ | 11.340 | 907 | 21.13 | C00183 | HMDB00883 | Valine, leucine and isoleucine degradation |
| 2-Aminobutyric acid | 11.380 | 826 | 14.18 | C02261 | HMDB39807 | ­- |
| Urea | 11.976 | 940 | 19.10 | C00086 | HMDB00294 | Arginine and proline metabolism |
| Leucine | 12.417 | 889 | 19.90 | C00123 | HMDB00687 | Valine, leucine and isoleucine biosynthesis |
| Phosphoric acid | 12.428 | 884 | 20.56 | C00009 | HMDB02142 | Oxidative phosphorylation |
| Glycerol | 12.454 | 940 | 10.44 | C00116 | HMDB00131 | Glycerolipid metabolism |
| Isoleucine^a^ | 12.809 | 918 | 15.85 | C00407 | HMDB00172 | Valine, leucine and isoleucine degradation |
| Proline^a^ | 12.893 | 877 | 20.07 | C00148 | HMDB00162 | Arginine and proline metabolism |
| Glycerate | 13.037 | 842 | 34.98^b^ | C00258 | HMDB00139 | Glycerolipid metabolism |
| Glyceric acid | 13.471 | 829 | 14.95 | C00258 | HMDB00139 | Glycerolipid metabolism |
| Methylmalonic acid | 13.813 | 801 | 17.66 | C02170 | HMDB00202 | Valine, leucine and isoleucine degradation |
| Fumarate^a^ | 13.882 | 905 | 19.65 | C00122 | HMDB00134 | Tricarboxylic acid cycle |
| Serine^a^ | 14.006 | 886 | 17.78 | C00065 | HMDB00187 | Glycine, serine and threonine metabolism |
| Threonine^a^ | 14.436 | 935 | 16.93 | C00188 | HMDB00167 | Glycine, serine and threonine metabolism |
| Aminomalonic acid | 15.847 | 765 | 32.73^b^ | C00872 | HMDB01147 | ­- |
| Pyroglutamic acid^a^ | 16.687 | 941 | 13.61 | C01879 | HMDB00267 | Glutathione metabolism |
| 2,3,4-Trihydroxybutyric acid | 17.233 | 864 | 11.10 | C05283 | HMDB11738 | ­- |
| Citrulline | 18.109 | 686 | 22.17 | C00327 | HMDB00904 | Arginine and proline metabolism |
| Glutamic acid^a^ | 18.181 | 835 | 13.88 | C00025 | HMDB00148 | Alanine, aspartate and glutamate metabolism |
| Phenylalanine^a^ | 18.285 | 898 | 14.22 | C00079 | HMDB00159 | Phenylalanine metabolism |
| Ribitol | 18.96 | 820 | 11.83 | C00474 | HMDB00508 | Pentose and glucuronate interconversions |
| L-Lysine^a^ | 19.445 | 889 | 25.96 | C00047 | HMDB00182 | Lysine degradation |
| Hypoxanthine | 20.811 | 915 | 15.71 | C00262 | HMDB00157 | Purine metabolism |
| Ornithine^a^ | 20.923 | 860 | 25.65 | C00077 | HMDB00214 | Arginine and proline metabolism |
| Citrate^a^ | 20.935 | 833 | 22.75 | C00158 | HMDB00094 | Citrate cycle |
| 1,5-Anhydro-sorbitol | 21.374 | 839 | 10.26 | C05145 | HMDB03911 | Pyrimidine metabolism |
| Fructose^a^ | 21.600 | 841 | 15.80 | C02336 | HMDB00660 | Amino sugar and nucleotide sugar metabolism |
| Galactose | 21.781 | 895 | 11.12 | C01582 | HMDB00143 | Glycolysis / Gluconeogenesis |
| Glucose ^a^ | 21.946 | 924 | 10.42 | C00031 | HMDB00122 | Glycolysis / Gluconeogenesis |
| Mannose | 22.177 | 963 | 10.07 | C00159 | HMDB00169 | Glycolysis / Gluconeogenesis |
| Tyrosine^a^ | 22.519 | 920 | 14.41 | C00082 | HMDB00158 | Tyrosine metabolism |
| Allonic acid | 22.705 | 793 | 14.81 | C00257 | ­- | Pentose phosphate pathway |
| α-D-Galactopyranose | 23.551 | 755 | 23.15 | C02267 | HMDB14692 | Glycolysis / Gluconeogenesis |
| Palmitelaidic acid | 23.627 | 861 | 9.09 | ­- | HMDB12328 | Fatty acid metabolism |
| Palmitic acid^a^ | 23.887 | 929 | 11.29 | C00249 | HMDB00220 | Fatty acid metabolism |
| Myo-Inositol | 24.296 | 862 | 16.00 | C00137 | HMDB00211 | Inositol phosphate metabolism |
| Uric acid | 24.444 | 827 | 22.24 | C00366 | HMDB00289 | Purine metabolism |
| Tryptophan | 25.782 | 892 | 21.79 | C00078 | HMDB00929 | Phenylalanine, tyrosine and tryptophan biosynthesis |
| Linoleic acid^a^ | 25.797 | 951 | 14.66 | C01595 | HMDB00673 | Biosynthesis of unsaturated fatty acids |
| Oleic acid^a^ | 25.864 | 941 | 11.03 | C00712 | HMDB00207 | Fatty acid biosynthesis |
| Stearic acid^a^ | 26.152 | 931 | 16.01 | C01530 | HMDB00827 | Fatty acid metabolism |
| Arachidonic acid^a^ | 27.487 | 818 | 22.16 | C00219 | HMDB01043 | Arachidonic acid metabolism |
| Glyceryl palmitate | 29.666 | 937 | 20.30 | ­- | HMDB31074 | Fatty acid metabolism |
| Glycerol monostearate | 31.461 | 932 | 20.03 | C15226 | HMDB13724 | Fatty acid metabolism |
| Cholesterol^a^ | 35.023 | 923 | 18.30 | C00187 | HMDB00067 | Primary bile acid biosynthesis |

a: 25 metabolites validated with standard sample; R-match: the matching score from NIST search; RSD: a relative standard deviation; b: the RSD value large than 30%; R-match: derived from a modified cosine of the angle between extracted mass spectrum and spectrum in library (normalized dot product). KEGG, HMDB: database ID (HMDB-human metabolome database); Pathways: the most relevant pathway analyzed by KEGG.
