# Supplementary material for: Characterizing semen abnormality male infertility using non-targeted blood plasma metabolomics
Source: PLoS One. 2019 Jul 5;14(7):e0219179. doi: 10.1371/journal.pone.0219179 (PMC6611580; doi:10.1371/journal.pone.0219179)
Supplement: S2 Table — (DOCX) [file pone.0219179.s002.docx]

S2 Table. Statistical analysis of age, smoking and drinking among SA subgroups and HC.

| **Statistical characteristics** | **TE (n = 21)** | **AS (n = 23)** | **OL (n = 20)** | **AZ (n = 20)** | **HC (n = 29)** | **p-value** |
| --- | --- | --- | --- | --- | --- | --- |
| Age (Avg±Std) | 28.98±4.80 | 28.85±5.38 | 28.78±5.62 | 28.60±4.93 | 28.07±4.51 | 0.967^a^ |
| Smoking (Yes/No) | 5/16 | 7/16 | 7/13 | 8/12 | 10/19 | 0.847^b^ |
| Drinking (Yes/No) | 0/21 | 0/23 | 0/20 | 0/20 | 0/29 | None^c^ |

a: p-value obtained by ANOVA with Dunnett post-hoc test; b: p-value obtained by Chi-Square Test; c: no result. No significant difference of drinking is obtained because all subjects of drinking are excluded before statistical analysis.
