# Supplementary material for: Characterizing semen abnormality male infertility using non-targeted blood plasma metabolomics
Source: PLoS One. 2019 Jul 5;14(7):e0219179. doi: 10.1371/journal.pone.0219179 (PMC6611580; doi:10.1371/journal.pone.0219179)
Supplement: S3 Table — (DOCX) [file pone.0219179.s003.docx]

S3 Table. The p-value of ANOVA with Dunnett post-hoc test.

| **Metabolites** | **p-value of ANOVA** | | | |
| --- | --- | --- | --- | --- |
|  | **TE-HC** | **AS-HC** | **OL-HC** | **AZ-HC** |
| Oxalic acid | 0.052 | 0.734 | 0.008 | 0.209 |
| Lactate | 0.999 | 0.065 | 0.010 | 0.937 |
| Alanine | 0.053 | 0.005 | 0.238 | 0.348 |
| Glycine | 0.083 | 0.576 | 0.880 | 0.000 |
| α-hydroxybutyrate | 0.993 | 0.968 | 1.000 | 0.838 |
| N-acetylglycine | 0.701 | 0.707 | 0.965 | 1.000 |
| β-hydroxybutyric acid | 0.697 | 0.645 | 0.845 | 0.993 |
| Valine | 1.000 | 0.831 | 0.887 | 0.269 |
| 2-Aminobutyric acid | 0.001 | 0.018 | 0.015 | 0.009 |
| Urea | 0.001 | 0.003 | 0.059 | 0.004 |
| Leucine | 0.998 | 0.940 | 0.867 | 0.595 |
| Phosphoric acid | 0.443 | 0.009 | 0.071 | 0.141 |
| Glycerol | 0.509 | 0.616 | 0.037 | 0.395 |
| Isoleucine | 0.992 | 1.000 | 0.999 | 0.624 |
| Proline | 0.061 | 1.000 | 0.779 | 0.353 |
| Glyceric acid | 0.536 | 1.000 | 0.645 | 0.648 |
| Methylmalonic acid | 0.070 | 0.339 | 0.972 | 0.349 |
| Fumarate | 0.122 | 0.499 | 0.972 | 0.300 |
| Serine | 0.882 | 0.990 | 0.968 | 0.069 |
| Threonine | 0.941 | 0.994 | 0.970 | 0.336 |
| Pyroglutamic acid | 0.008 | 0.201 | 0.615 | 0.000 |
| 2,3,4-Trihydroxybutyric acid | 0.556 | 0.835 | 1.000 | 0.245 |
| Citrulline | 0.993 | 0.999 | 0.829 | 0.309 |
| Glutamic acid | 0.962 | 0.951 | 0.439 | 0.857 |
| Phenylalanine | 0.904 | 0.988 | 0.968 | 0.951 |
| Ribitol | 0.908 | 1.000 | 0.258 | 0.999 |
| L-Lysine | 0.092 | 0.167 | 0.565 | 0.578 |
| Hypoxanthine | 1.000 | 0.939 | 0.198 | 0.771 |
| Ornithine | 0.004 | 0.003 | 0.012 | 0.002 |
| Citrate | 0.001 | 0.002 | 0.027 | 0.000 |
| 1,5-Anhydro-sorbitol | 0.001 | 0.002 | 0.001 | 0.002 |
| Fructose | 0.992 | 0.975 | 0.194 | 0.710 |
| Galactose | 0.202 | 0.002 | 0.005 | 0.052 |
| Glucose | 0.814 | 1.000 | 0.589 | 0.603 |
| Mannose | 0.991 | 0.978 | 0.323 | 0.883 |
| Tyrosine | 0.977 | 0.960 | 0.849 | 0.982 |
| Allonic acid | 0.997 | 0.503 | 0.176 | 0.997 |
| α-D-Galactopyranose | 0.500 | 0.147 | 0.018 | 0.578 |
| Palmitelaidic acid | 0.305 | 1.000 | 0.573 | 0.520 |
| Palmitic acid | 0.896 | 0.976 | 0.982 | 0.675 |
| Myo-Inositol | 0.119 | 0.135 | 0.023 | 0.063 |
| Uric acid | 0.980 | 0.914 | 0.196 | 0.305 |
| Tryptophan | 0.921 | 0.985 | 0.990 | 0.375 |
| Linoleic acid | 0.252 | 0.547 | 0.548 | 0.554 |
| Oleic acid | 0.860 | 0.987 | 0.988 | 0.995 |
| Stearic acid | 0.352 | 0.194 | 0.291 | 0.596 |
| Arachidonic acid | 0.167 | 0.927 | 0.711 | 0.120 |
| Glyceryl palmitate | 1.000 | 0.000 | 0.000 | 1.000 |
| Glycerol monostearate | 1.000 | 0.000 | 0.000 | 1.000 |
| Cholesterol | 1.000 | 0.909 | 0.928 | 0.999 |
