# Supplementary material for: Characterizing semen abnormality male infertility using non-targeted blood plasma metabolomics
Source: PLoS One. 2019 Jul 5;14(7):e0219179. doi: 10.1371/journal.pone.0219179 (PMC6611580; doi:10.1371/journal.pone.0219179)
Supplement: S4 Table — (DOCX) [file pone.0219179.s004.docx]

S4 Table. The VIP value of PLS-DA models.

| **Metabolites** | **VIP value of PLS-DA model** | | | |
| --- | --- | --- | --- | --- |
|  | **TE-HC** | **AS-HC** | **OL-HC** | **AZ-HC** |
| Oxalic acid | 1.533 | 0.549 | 1.225 | 0.922 |
| Lactate | 0.615 | 1.488 | 1.395 | 0.826 |
| Alanine | 1.352 | 1.671 | 0.927 | 0.966 |
| Glycine | 1.417 | 0.767 | 0.692 | 1.819 |
| α-hydroxybutyrate | 0.410 | 0.391 | 0.479 | 0.664 |
| N-acetylglycine | 0.742 | 0.706 | 0.812 | 0.535 |
| β-hydroxybutyric acid | 0.722 | 0.680 | 0.556 | 0.506 |
| Valine | 0.423 | 0.513 | 0.535 | 1.020 |
| 2-Aminobutyric acid | 1.823 | 1.403 | 1.186 | 1.269 |
| Urea | 1.826 | 1.782 | 0.988 | 1.472 |
| Leucine | 0.551 | 0.453 | 0.582 | 1.007 |
| Phosphoric acid | 0.776 | 1.468 | 0.947 | 0.936 |
| Glycerol | 0.703 | 0.570 | 1.064 | 0.782 |
| Isoleucine | 0.568 | 0.292 | 0.499 | 0.933 |
| Proline | 1.131 | 0.363 | 0.735 | 1.059 |
| Glyceric acid | 0.823 | 0.350 | 0.629 | 0.964 |
| Methylmalonic acid | 1.263 | 0.930 | 0.523 | 0.798 |
| Fumarate | 1.189 | 0.819 | 0.527 | 0.823 |
| Serine | 1.054 | 0.604 | 1.079 | 1.204 |
| Threonine | 1.025 | 0.651 | 0.846 | 0.901 |
| Pyroglutamic acid | 1.547 | 0.996 | 0.863 | 1.656 |
| 2,3,4-Trihydroxybutyric acid | 0.777 | 0.521 | 0.286 | 0.950 |
| Citrulline | 0.629 | 0.196 | 0.619 | 0.845 |
| Glutamic acid | 0.323 | 0.423 | 0.866 | 0.836 |
| Phenylalanine | 0.888 | 0.635 | 0.716 | 1.015 |
| Ribitol | 0.511 | 0.458 | 0.868 | 0.631 |
| L-Lysine | 1.395 | 1.162 | 0.765 | 1.090 |
| Hypoxanthine | 0.152 | 0.278 | 0.767 | 0.475 |
| Ornithine | 1.431 | 1.373 | 0.968 | 1.471 |
| Citrate | 1.941 | 1.809 | 1.391 | 1.795 |
| 1,5-Anhydro-sorbitol | 1.786 | 1.614 | 1.538 | 1.532 |
| Fructose | 0.582 | 0.871 | 0.762 | 0.660 |
| Galactose | 0.946 | 1.518 | 1.253 | 1.362 |
| Glucose | 0.563 | 0.301 | 0.816 | 0.755 |
| Mannose | 0.253 | 0.326 | 0.886 | 0.635 |
| Tyrosine | 0.759 | 0.475 | 0.629 | 0.892 |
| Allonic acid | 0.209 | 0.649 | 0.918 | 0.592 |
| α-D-Galactopyranose | 0.721 | 0.937 | 1.146 | 0.892 |
| Palmitelaidic acid | 0.949 | 0.405 | 0.827 | 0.823 |
| Palmitic acid | 0.640 | 0.589 | 0.850 | 0.814 |
| Myo-Inositol | 0.912 | 0.920 | 1.003 | 0.954 |
| Uric acid | 0.238 | 0.358 | 0.795 | 1.112 |
| Tryptophan | 0.443 | 0.416 | 0.510 | 0.985 |
| Linoleic acid | 0.994 | 0.730 | 0.774 | 0.680 |
| Oleic acid | 0.859 | 0.625 | 0.964 | 0.821 |
| Stearic acid | 0.964 | 1.208 | 1.118 | 0.790 |
| Arachidonic acid | 1.081 | 0.441 | 0.769 | 1.073 |
| Glyceryl palmitate | 0.453 | 2.400 | 2.514 | 0.359 |
| Glycerol monostearate | 1.195 | 2.416 | 2.530 | 0.973 |
| Cholesterol | 0.621 | 0.571 | 0.429 | 0.379 |
