# Supplementary figures and images for: Characterizing semen abnormality male infertility using non-targeted blood plasma metabolomics
Source: PLoS One. 2019 Jul 5;14(7):e0219179. doi: 10.1371/journal.pone.0219179 (PMC6611580; doi:10.1371/journal.pone.0219179)

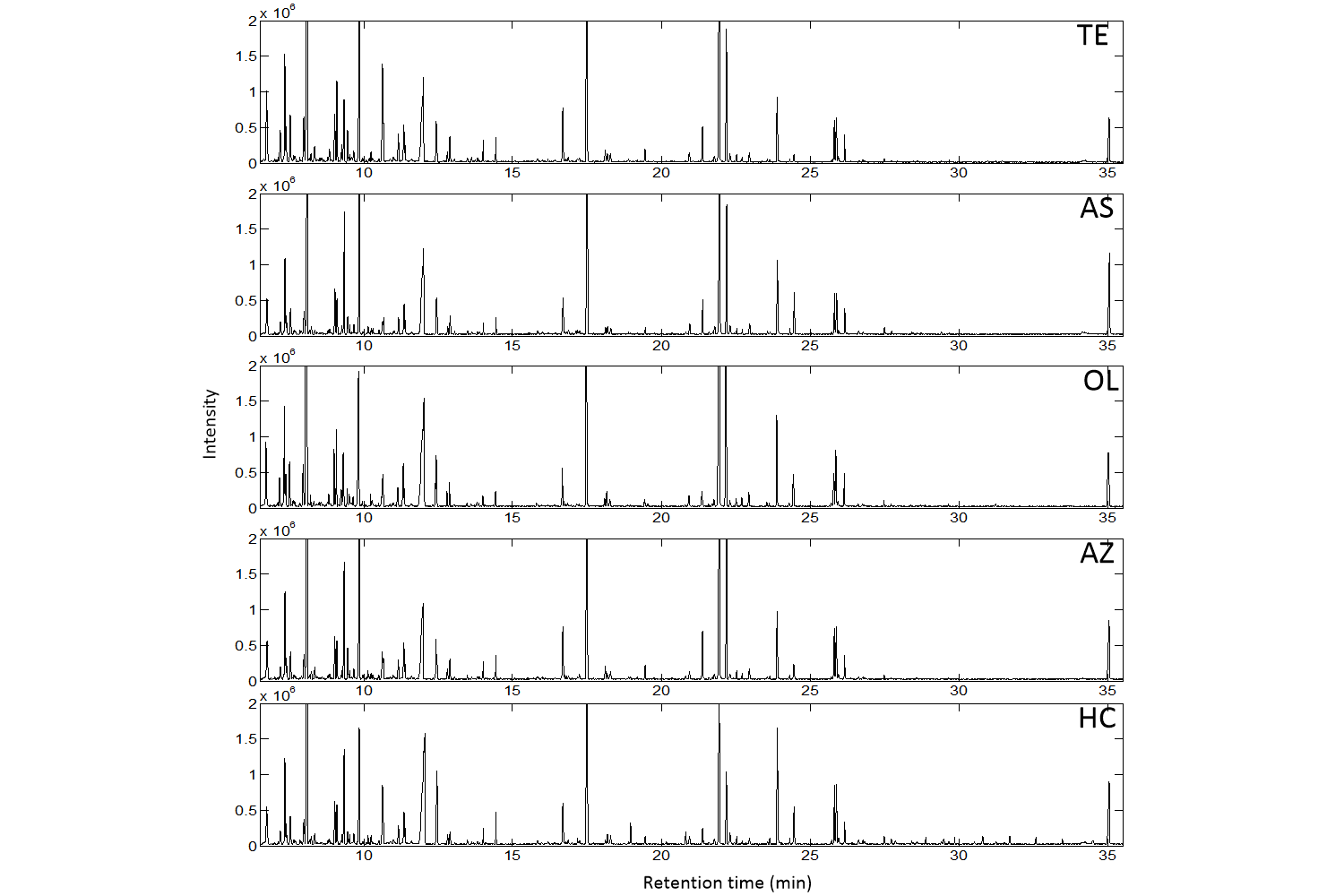

Supplement: S1 Fig — (TIF) [file pone.0219179.s005.tif]

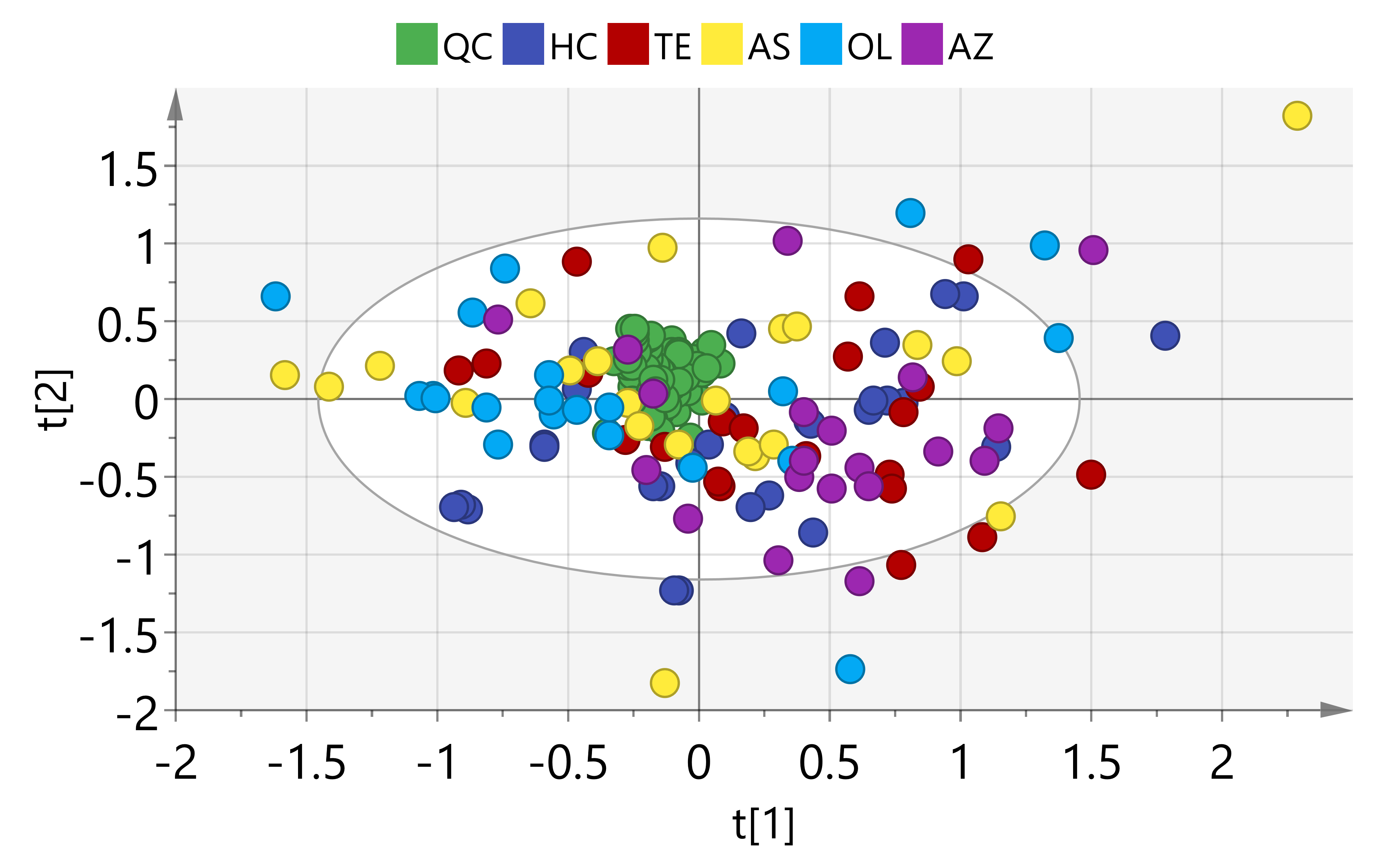

Supplement: S2 Fig — (TIF) [file pone.0219179.s006.tif]

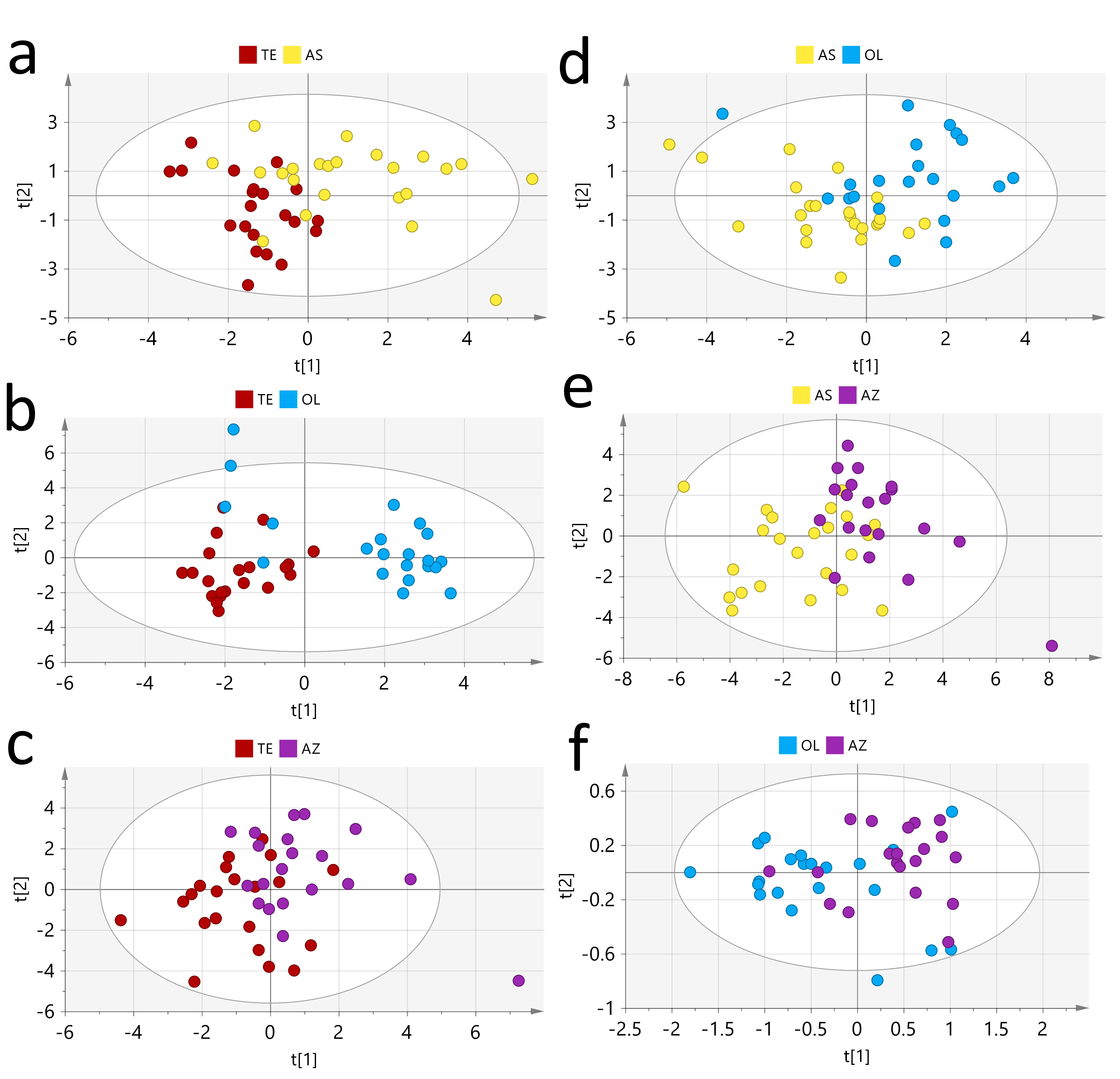

Supplement: S3 Fig — The R2X, R2Y and Q2 of six models: a, 0.197, 0.513, -0.167; b, 0.253, 0.722, 0.234; c, 0.258, 0.399, -0.21; d, 0.241, 0.398, 0.047; e, 0.284, 0.492, 0.071; f, 0.323, 0.334, 0.131. (TIF) [file pone.0219179.s007.tif]
